# The Book World of Medicine and Science

**Published:** 1905-07-22

**Authors:** 


### The Book World of Medicine and Science.

Carcinoma of the Rectum. By F. Swinford Edwards, F.R.C.S. (London: Baillière, Tindall and Cox. 1905. Pp. 47. Price 2s. 6d. net.)

This little book embodies the author's personal experience of carcinoma of the rectum, and, as is usual with such works, it is very pleasant and instructive to read. Probably some readers will be disappointed that Mr. Edwards has not entered more into detail, especially where he describes the technique of operative procedures; however, simplicity is an admirable defect. The most interesting feature of the book is the author's strong advocacy of inguinal colotomy as a preliminary to removal of a new growth in the rectum. He advises that the colotomy should be performed about a fortnight before the radical operation, and does not consider the delay which is thus caused to be of serious importance. With regard to colotomy as a palliative procedure in cases where removal of the growth is not possible, Mr. Edwards strongly recommends the operation as soon as the symptoms of the disease have become so marked as to interfere with the comfort of the patient. The book concludes with a table showing the results of operation in a number of cases which have been under the author's care.

An Introduction to Midwifery. By Archibald Donald, M.A., M.D. (London: Charles Griffin and Co., Limited. Price 5s.)

This is the sixth edition of the above work. It shows no new features except the addition of some extracts from the rules of the Central Midwives Board, and a revision in accordance with the Act of those parts of the book which more especially apply to midwives.

What Venereal Diseases Mean and How to Prevent Them. By Professor Erik Pontoppidan. (London: John Bale, Sons, and Danielsson. Pp. 79. Price 2s. net.)

This brochure comprises the substance of some lectures delivered by Professor Pontoppidan to the students at the University of Copenhagen. The author's chief aims are to impress students with the very serious evils which may follow venereal infection and thus to overcome the frivolity with which the latter is sometimes regarded by certain classes of students. He refers in the briefest manner to therapeutic measures of prevention, and regards open-air sports and exercises as the most potent aids towards good moral tone and the chief agents in encouraging natural prophylaxis against venereal infection. We have nothing but commendation for the author's views and the tactful manner in which he has presented them.

"Verb. Sap." on Going to West Africa, to Northern and Southern Nigeria, and to the Coasts. By Allan Field, F.R.G.S. (London: Bale, Sons, and Danielsson. Price 2s. 6d.)

This is a well classified, "handy" book for all intending travellers to the West Coast of Africa. There is little superfluous knowledge to perplex the uninitiated, but the smallest necessary details are contained in it. The writer gives sound advice regarding the treatment of the natives, dwelling upon the necessity to pay due respect to their customs. He has allowed a generous margin to the outfit department, very essential in such a climate; but the individual traveller would of necessity reduce the list considerably. We can endorse his advice as regards the arrangement of mosquito netting and the observance of sanitary precautions, although the little guide is written in a style somewhat more discursive than is usual in a practical work of the kind. The writer has introduced much information and originality of observation which render the book interesting even to the casual reader who may never put the guide to practical use.

Herbert Fry's Royal Guide to the London Charities, 1905. (London: Chatto and Windus, 111 St. Martin's Lane. Price 1s. 6d.)

This is an alphabetically-arranged directory in ruled columns. This arrangement is convenient but it limits the scope of the information which in this handbook is consequently slight. It consists of the name and address of the institution, the date of its foundation, its objects, receipts, number of persons benefited, where to apply for information and the names of some of the officials. A glance at the financial columns reveals the incomplete nature of the information it affords, and it is difficult to see what useful purpose is served by its inclusion. To render a book of the kind so exhaustive and accurate as to be of practical utility is a very expensive matter, as anyone who has been engaged upon the compilation of books of reference knows. Too much therefore should not be expected of a work of so small a cost. It contains a classified index.

Low's Handbook to the Charities of London, 1905. (London: Leighton House, Fleet Street. Price 1s.)

This is the 70th year of publication of this little list of charities. The compilation is alphabetical, and the index is classified. It is intended that the information should be uniform and it is stated to have been obtained from the latest reports. The authorities of charitable institutions find it difficult to co-operate with compilers of the many lists extant, and there are very numerous evidences in Low's Handbook to show that the editor has been unable to give up-to-date figures, probably for this reason.

Baillière, Tindall and Cox.

"Manual of Midwifery." By Hy. Jellett, M.D.

"Carcinoma of the Rectum." By F. Swinford Edwards, F.R.C.S.

"Handbook of Intestinal Surgery." By L. A. Bidwell, F.R.C.S.

Homœopathic Publishing Company.

"Homœopathy Explained." By J. H. Clarke, M.D.

"Low's Handbook to the Charities of London, 1905.

